# Production of Betacyanins in Transgenic *Nicotiana tabacum* Increases Tolerance to Salinity

**DOI:** 10.3389/fpls.2021.653147

**Published:** 2021-04-30

**Authors:** Yanfei Zhou, Tanja Karl, David H. Lewis, Tony K. McGhie, Steve Arathoon, Kevin M. Davies, Ken G. Ryan, Kevin S. Gould, Kathy E. Schwinn

**Affiliations:** ^1^The New Zealand Institute for Plant and Food Research Limited, Palmerston North, New Zealand; ^2^School of Biological Sciences, Victoria University of Wellington, Wellington, New Zealand

**Keywords:** *Nicotiana tabacum*, betacyanin, betalain, photoprotection, salinity, antioxidant

## Abstract

Although red betalain pigments (betacyanins) have been associated with salinity tolerance in some halophytes like *Disphyma australe*, efforts to determine whether they have a causal role and the underlying mechanisms have been hampered by a lack of a model system. To address this, we engineered betalain-producing *Nicotiana tabacum*, by the introduction of three betalain biosynthetic genes. The plants were violet-red due to the accumulation of three betacyanins: betanin, isobetanin, and betanidin. Under salt stress, betacyanic seedlings had increased survivability and leaves of mature plants had higher photochemical quantum yields of photosystem II (*F*_*v*_/*F*_*m*_) and faster photosynthetic recovery after saturating light treatment. Under salt stress, compared to controls betacyanic leaf disks had no loss of carotenoids, a slower rate of chlorophyll degradation, and higher *F*_*v*_/*F*_*m*_ values. Furthermore, simulation of betacyanin pigmentation by using a red filter cover improved *F*_*v*_/*F*_*m*_ value of green tissue under salt stress. Our results confirm a direct causal role of betacyanins in plant salinity tolerance and indicate a key mechanism is photoprotection. A role in delaying leaf senescence was also indicated, and the enhanced antioxidant capability of the betacyanic leaves suggested a potential contribution to scavenging reactive oxygen species. The study can inform the development of novel biotechnological approaches to improving agricultural productivity in saline-affected areas.

## Introduction

Salt-tolerant plants are able to grow and complete their life cycle on a substrate that contains high concentrations of soluble salt. Plants have developed a wide spectrum of mechanisms to adapt to salt stress, including regulating ion uptake and compartmentalization (selective accumulation or exclusion, compartmentalization in cells or organs, control of uptake by roots), osmotic adjustment (accumulation of compatible solutes), enhancing antioxidant capacity (free radical scavenging), hormone modulation (whole metabolism regulation), and stomatal closure (increasing water use efficiency) (Hasegawa et al., 2000; Parida and Das, 2005; Munns and Tester, 2008; Krasensky and Jonak, 2012; van Zelm et al., 2020).

Recent work suggests that red plant pigments can also play an important role in salt tolerance (Polturak et al., 2018). Concentrations of anthocyanin, a type of red pigment commonly occurring in plants, often increase substantially in vegetative shoots exposed to salt stress, and this has been associated with improved salinity tolerance. Salt treatment increased anthocyanin accumulation in wheat leaves, and wheat genotypes with high anthocyanin content were able to maintain significantly higher dry matter production after salt stress treatment (Mbarki et al., 2018). Ectopic expression of an *Arabidopsis thaliana* uridine diphosphate (UDP) glycosyltransferase (UGT79B2/B3) significantly increased anthocyanin concentration and enhanced salt tolerance, with increased plant antioxidant capacity thought to be the mechanism (Li et al., 2017). Similarly, overexpression of the *Antirrhinum majus* anthocyanin pathway transcriptional activators *Delila* or *Rosea1* enhanced anthocyanin production in *Nicotiana tabacum* leaves and flowers, which improved salt tolerance by augmenting radical-scavenging activities (Naing et al., 2017, 2018).

Betalains are a group of water-soluble pigments that include the red betacyanins and the yellow betaxanthins. In plants, they occur solely among certain families in the Caryophyllales, where they are considered to replace the anthocyanins. Their base moiety is derived from tyrosine and the core pathway is comprised of only a few enzymatic steps (Supplementary Figure 1), the genes for which have been identified (Strack et al., 2003; Christinet et al., 2004; Sasaki et al., 2005; Hatlestad et al., 2012; Polturak et al., 2016; Polturak and Aharoni, 2018). The accumulation of betacyanin pigments in vegetative tissue under salt stress has been observed in a range of species including *Amaranthus hypochondriacus*, *Portulaca oleracea*, *Alternanthera philoxeroides*, and *Mesembryanthemum crystallinum* (Casique-Arroyo et al., 2014; Ribeiro et al., 2014; Mulry et al., 2015; Oh et al., 2015), and has been correlated with induction of betalain biosynthetic gene expression (Casique-Arroyo et al., 2014). One of the most studied species is *Disphyma australe*, a member of the Aizoaceae (iceplant) family. The red coloration in *D. australe* results from the production of betacyanin (Chinnock, 1971). In *D. australe* “red” and “green” vegetative morphs exist naturally, with the red morphs being more salt tolerant (Jain and Gould, 2015; Jain et al., 2015). Furthermore, the induction of betacyanins in the green morph by feeding the pathway intermediate L-DOPA reduced the detrimental effects of salt treatment, suggesting the benefit is caused specifically by betacyanin production (Jain et al., 2015).

The mechanism(s) through which betacyanins help with salt tolerance are not fully understood. Given the capacities of these red pigments to absorb green and yellow quanta, they should be able to effectively reduce excitation pressure on chloroplasts for which the light harvesting properties have been compromised by salt stress. Accordingly, the *D. australe* red morph shows less evidence of photo-oxidative damage than does the green one, and induced betacyanin production in the green morph provided photoprotection during salt stress (Jain and Gould, 2015; Jain et al., 2015). Photoprotection from anthocyanins has also been implicated in enhancing salt tolerance (Narbona et al., 2018; Zheng et al., 2019; Lo Piccolo et al., 2020).

Salt stress causes over production of reactive oxygen species (ROS), which leads to cellular damage and even plant cell death. Betacyanins are noted for their strong antioxidant properties in *in vitro* assays (Belhadj Slimen et al., 2017), and may have a role *in planta* as ROS scavengers during stress conditions. However, at least for *D. australe*, the accumulation of the pigments is restricted to the epidermis, arguing against such a role in salt stress responses in this species (Jain et al., 2015).

Recently, researchers have successfully transferred the genes required for betacyanin biosynthesis into non-betacyanic species (Polturak et al., 2016, 2017), allowing model systems to be developed to test causative roles for betacyanin pigments in abiotic and biotic stress tolerance. To further investigate the proposed contribution of betacyanin pigmentation to salt stress tolerance, we produced betacyanic *N. tabacum*, a species that does not naturally produce these pigments. We hypothesized that: (1) the benefit that betacyanins confer for plant salt tolerance could be transferred to non-betacyanic plants; (2) the production of betacyanin would deliver photoprotection and ROS scavenging benefit to plants under salt stress.

We demonstrate the beneficial effect of betacyanins on *N. tabacum* photosynthesis protection, ROS scavenging capability and seedling survival under salt stress. This study also illustrates a new approach for conferring increased salt tolerance to plants, which may prove useful for generating more resilient crop plants.

## Materials and Methods

### Plant Material and Greenhouse Conditions

The *N. tabacum* plants (cultivar: Samsun) were geminated from seeds and grown in pots (85 mm × 85 mm × 100 mm) in a greenhouse in Palmerston North, New Zealand. The greenhouse was heated at 15°C and vented at 25°C, with natural lighting. Plants were watered with tap water until salt treatment started.

### Binary Vector Construction

**FIGURE 1:**
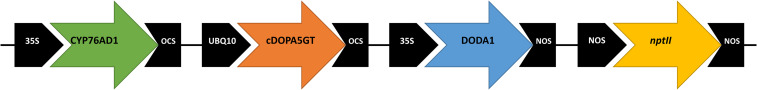
Schematic of vector pYF1 used for overexpression of the betalain biosynthetic genes CYP76AD1 (*B. vulgaris* cytochrome P450, GenBank HQ656023.1), cDOPA5GT (*Mirabilis jalapa cyclo*-DOPA-5-*O*-glucosyltransferase, AB182643.1), and DODA1 (*B. vulgaris* DOPA 4,5-dioxygenase, HQ656027.1). The vector included the *nptII* kanamycin resistance selectable marker.

Gene sequences used in this study were for BvCYP76AD1 (GenBank accession HQ656023.1), MjcDOPA5GT (AB182643.1) and BvDODA1 (HQ656027.1). The CYP76AD1, cDOPA5GT, and BvDODA1 open reading frame cDNA sequences were located, respectively, between the *35SCaMV* promoter and the *octopine synthase* (*OCS*) terminator, the Arabidopsis *ubiquitin 10* (*UBQ10*) promoter and *OCS* terminator, and the *35SCaMV* promoter and the *nopaline synthase* (*NOS*) terminator sequences (Figure 1). The fragment containing the three transgenes was synthesized by GenScript^1^, and then ligated into the pART27 vector (Gleave, 1992), which contains a kanamycin selectable marker gene, resulting in the binary vector pYF1.

### Plant Transformation and Regeneration

Stable transgenic *N. tabacum* plants harboring the T-DNA regions from pYF1 or empty pART27 were generated by *Agrobacterium tumefaciens*–mediated (strain GV3101) leaf-disk transformation, essentially as described in Horsch et al. (1985). Wild type plants were regenerated from explants through the same process as the transgenic lines. The different plant lines are referred to as wild type (WT), empty vector control (EV), and betalain-overexpression (BtOE).

### Leaf Disk Assay

The leaf disk assay described by Sanan-Mishra et al. (2005) was conducted with minor modification. Four independent lines of each type of transgenic plant were used. To generate sufficient leaf disks for all treatments, three clonal plants of each independent transgenic line (T0) and WT (regenerated through tissue culture) (8 weeks old) were used. The third mature leaf (healthy and fully expanded) was collected from each plant. Leaf disks of 1.8-cm diameter were excised from the central portion of the lamina either side of the midrib. For each treatment, one leaf disk from four independent lines of each type of plant was used. The disks were floated on 5 mL of NaCl solution at 100 mM or 200 mM, or on distilled water (experimental control) for 48 h at 22°C under white light (150 or 450 μmol m^–2^ s^–1^) provided by a cool white LED panel with a 12 h photoperiod. Wild type *N. tabacum* leaf disk treated with 100 mM or 200 mM NaCl for 3 days in a leaf disk senescence assay showed mild and severe senescence, respectively, (Sanan-Mishra et al., 2005), so this concentration was used in salt stress tests. Pigment content was measured on each leaf disk after the treatment.

To simulate the light filter effect of betacyanins, another set of WT and EV leaf disks floated on the same concentration of NaCl solution was covered by a red polycarbonate filter (Rosco Supergel #346 Tropical Magenta, KEL-LPS, Auckland, New Zealand) with a similar absorption spectrum to betacyanins (530–550 nm) (Azeredo, 2009). The maximum quantum efficiency of photosystem II (*F*_*v*_/*F*_*m*_) was determined on each leaf disk after treatment using a Walz 2500 (Effeltrich, Germany) pulse amplitude modulated fluorometer (PAM) according to the manufacturer’s operating instructions^2^.

### Photo Recovery Assay

T0 transgenic and WT seedlings were generated from tissue culture as described in “Plant transformation and regeneration” and then grown in pots (85 mm × 85 mm × 100 mm) in the greenhouse as described above, for 2 months. Four independent lines of each type of transgenic plant were used. Plants were irrigated daily for 2 weeks with 50 mL of tap water or 400 mM NaCl. Leaves of a similar size and age were used to monitor chlorophyll fluorescence before, during, and after treatment with saturating light. Maximum quantum efficiency of photosystem II (*F*_*v*_/*F*_*m*_) was determined for one leaf per plant. Leaves were dark adapted for 30 min before taking the initial *F*_*v*_/*F*_*m*_ measurements. The leaves were then treated with 1000 μmol m^–2^ s^–1^ cool white light for 30 min and *F*_*v*_/*F*_*m*_ measurements were taken again. The plants were then returned to darkness and further *F*_*v*_/*F*_*m*_ measurements taken every hour for 5 h.

### Betalain Analysis

Fresh leaf tissue (100 mg) was ground into fine powder in liquid nitrogen and betalains were extracted with 2 mL methanol:water:formic acid (80:19:1). Ultra High Performance Liquid Chromatography (UHPLC) was used to separate the betalains. Liquid chromatography – High Resolution Accurate Mass – mass spectrometry (LC-HRAM-MS and LC-HRAM-MS/MS) was used to assist in assigning compound identities.

The UHPLC system used a Dionex Ultimate 3000^®^, Rapid Separation LC system equipped with a binary pump (HPR3400RS), autosampler (WPS-3000RS), column compartment (TCC-3000RS), and a diode array detector (DAD-3000RS). The analytical column was a Luna Omega C18 100 mm × 2.1 mm, 1.6 μm (Phenomenex, Torrance, CA, United States), maintained at 40°C. A binary solvent program was used with Solvent A (0.1% formic acid) and Solvent B (acetonitrile) at a flow of 300 μL min^–1^. The initial solvent composition was 100% A 0–0.5 min; linear gradient to 85% A 15% B 0.5–10 min; linear gradient to 40% A 60% B 10–20 min; linear gradient to 5% A 95% B 20–22 min; composition held at 5% A 95% B 22–25 min; linear gradient to 100% A 25–25.5 min; to return to the initial conditions before another sample injection at 30 min. The injection volume was 2 μL. Spectral data (260–600 nm) were collected for the entire analysis.

The LC-HRAM-MS/MS system was composed of a Dionex Ultimate 3000^®^, Rapid Separation LC and a micrOTOF QII high resolution mass spectrometer (Bruker Daltonics, Bremen, Germany) fitted with an electrospray ion source. The LC column was a Luna Omega C18 100 mm × 2.1 mm, 1.6 μm (Phenomenex, Torrance, CA, United States) and was maintained at 40°C. The flow was 300 μL min^–1^. The solvents were *A* = 0.2% formic acid and *B* = 100% acetonitrile. The solvent gradient was the same as for the UHPLC. The injection volume for samples and standards was 1 μL. The micrOTOF QII parameters were: temperature 225°C; drying N_2_ flow 6 L min^–1^; nebulizer N_2_ 1.5 bar, endplate offset 500 V, mass range 100–1500 Da, and data were acquired at 5 scans s^–1^. Positive ion electrospray was used with a capillary voltage of 3000 V. Post-acquisition internal mass calibration used sodium formate clusters with the sodium formate delivered by a syringe pump at the start of each chromatographic analysis. Data were processed using Target Analysis for Screening and Quantitation software (TASQ) (Bruker Daltonics, Bremen, Germany).

### Carotenoid and Chlorophyll Analysis

Chlorophylls and carotenoids were extracted from leaf tissue in 80% (v/v) acetone and measured spectrophotometrically using a Microplate Reader (SpectraMax Plus 384, Molecular Devices, CA, United States) according to the method described in Lichtenthaler and Buschmann (2001).

### Seedling Survival Assay

Two week-old seedlings (T2) of three independent lines of WT, EV, and BtOE plants were transferred to Murashige and Skoog (1962) (MS) medium (Sigma-Aldrich, St. Louis, MO, United States) containing 800 mM NaCl. Three technical replicates of eight seedlings each were grown for 8 days at 22°C under white light (450 μmol m^–2^s^–1^) provided by cool white LEDs, with a photoperiod of 12 h. The seedlings were then transferred to MS medium containing no NaCl for a recovery period of 2 weeks.

### Antioxidant Activity Measurement

T2 transgenic and WT plants were grown from seeds in pots (85 mm × 85 mm × 100 mm) in the greenhouse for 2 months. Four independent lines of each type of transgenic plant were used. Plants were irrigated daily with 50 mL of tap water or 400 mM NaCl for 5 days. The third mature leaf was collected from each plant after treatment. A leaf disk of 1.8-cm diameter (around 66 mg fresh weight) was excised from the central portion of each leaf lamina for total antioxidant activity quantification, and the rest of the leaf tissue was used for measurement of antioxidant or salt tolerance-related gene expression. The leaf disks were ground to a powder in liquid nitrogen and extracted with 1 mL 80% (v/v) methanol. Antioxidant capability was measured using the ABTS (2,2′-azino-*bis*(3-ethylbenzothiazoline-6-sulfonic acid) assay reported in Lim et al. (2016).

### Statistical Analyses

Reported data represent the means of at least three biological replicates, and are given ± standard errors. For statistical analysis, R (R Development Core Team, 2019) and the emmeans package were used. Relative carotenoid and chlorophyll concentration and *F*_*v*_/*F*_*m*_ measurements from leaf disks, seedling survival rate as well as photoinhibition and recovery were each analyzed using one-way ANOVA. Pairwise comparisons using the emmeans function were performed for comparisons across treatments.

## Results

### Generation of Betacyanin Producing *N. tabacum* by Expression of Betalain Biosynthesis Genes

**FIGURE 2:**
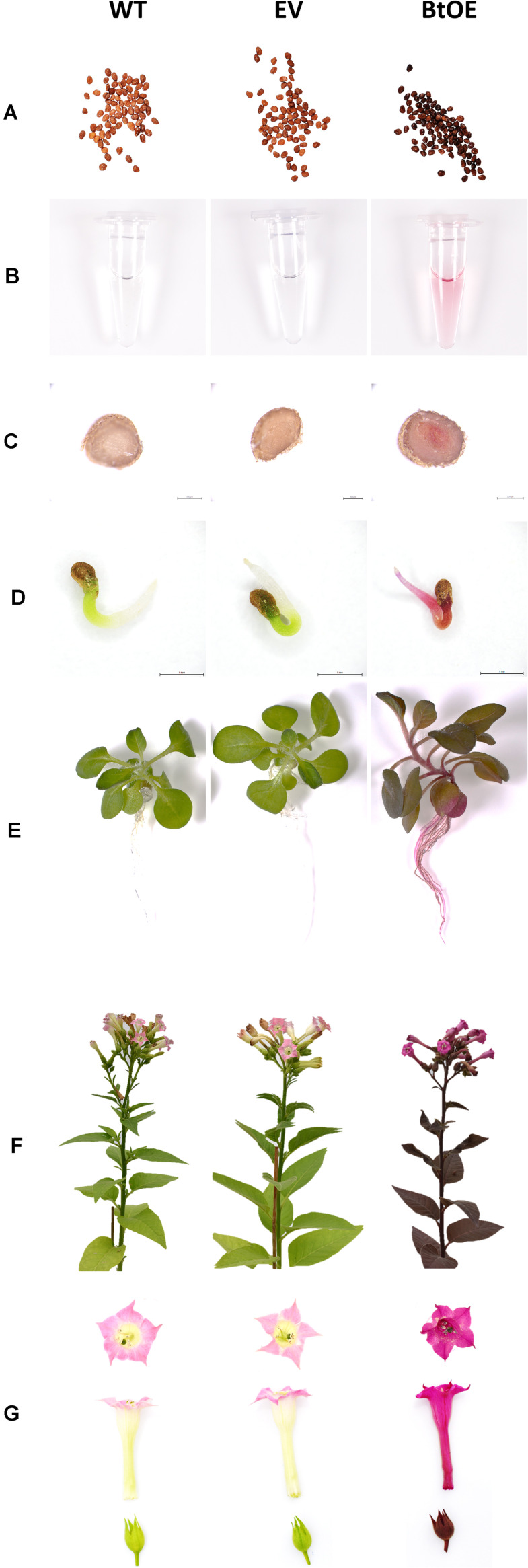
Introduction of the betalain overexpression vector to *Nicotiana tabacum* produces violet-red pigmentation in transgenic plants. Plant lines are wild type (WT), empty vector control (EV), and betacyanin overexpression transgenics (BtOE). **(A)** T3 homozygous seeds; **(B)** extract of seeds using 80% (v/v) methanol; **(C)** cross section of T3 homozygous seeds; **(D)** germinated seeds; **(E)** 4-week-old seedlings; **(F)** mature plants; **(G)** flowers.

**FIGURE 3:**
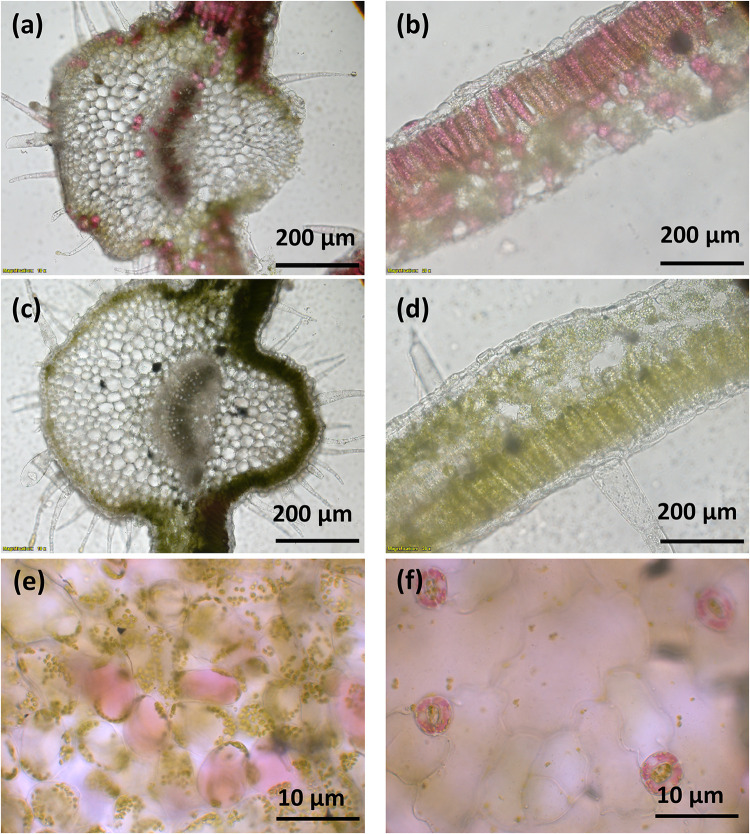
Betacyanin pigmentation in *Nicotiana tabacum*. Images **(a–d)** are of leaf cross sections from BtOE **(a,b)** and WT **(c,d)** *N. tabacum* plants. Images **(e,f)** show the mesophyll and guard cells, respectively, of BtOE plants.

**FIGURE 4:**
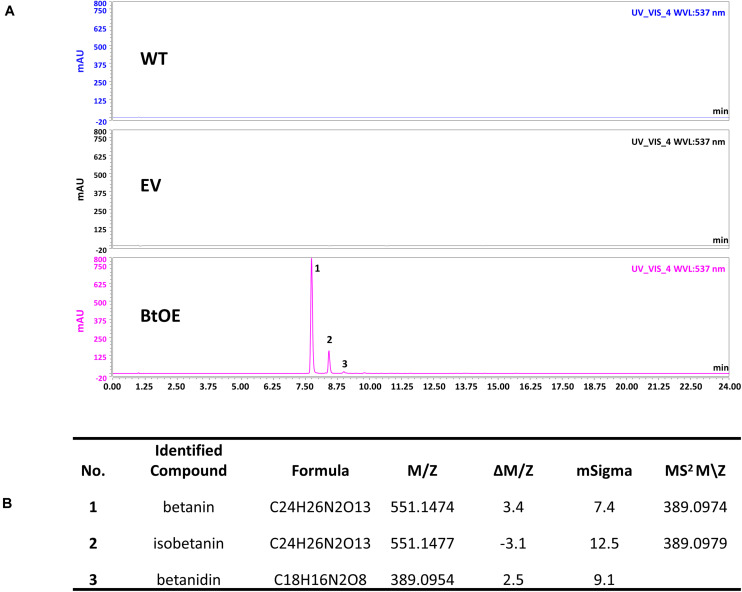
Identification of betacyanins in *N. tabacum*. **(A)** HPLC chromatogram of *N. tabacum* leaf extract. The horizontal axis indicates the retention time (min), whereas the vertical axis indicates the signal intensity (l V); **(B)** Betalains identified by LC-MS analysis.

A vector (pYZ1) harboring three betalain biosynthetic genes (*CYP76AD1*, *cDOPA5GT*, and *DODA1*) (Figure 1) was used to transform *N. tabacum*, which normally does not produce red pigmentation in its leaves. Betalain-overexpression (BtOE) plants had strong ectopic or enhanced red pigmentation in a range of tissues (Figures 2, 3), and analysis of leaf tissue confirmed transgene expression (Supplementary Figure 2) and betacyanins (betanin, isobetanin, and betanidin) as the basis of the red pigmentation (Figure 4). Three types of betaxanthins was also found in BtOE plants (Supplementary Figure 3), but their concentrations were comparatively very low, at trace amounts. No change was apparent in the *N. tabacum* phenylpropanoid profile from introduction of the betalain gene vector (Supplementary Figure 4). BtOE seeds were darker than seeds of wild type (WT) and empty vector control (EV) plants (Figure 2A), and extracts with 80% methanol showed a red color (Figure 2B). Cross sections of the BtOE seeds confirmed the presence of red pigments in the embryos (Figure 2C), and the cotyledon and radicle of the germinated seeds had a red-violet hue (Figure 2D). Four weeks after germination, the entire BtOE seedling showed strong red coloration, including leaves, stem, and root (Figure 2E). The production of red pigment did not obviously affect plant growth and development, and the plants flowered at the same age as WT (Figure 2F). BtOE flowers had a violet color and deeper pigmentation that extended further along the tube of the corolla than control flowers (which were weakly colored by anthocyanin pigments) (Figure 2G). In BtOE leaves, betacyanin pigments were abundant throughout leaf tissue, mainly accumulating in palisade and spongy mesophyll cells, cells around the vascular bundle, and in guard cells in the epidermis (Figure 3).

### Betacyanin Pigmentation Delayed Leaf Senescence Under Salt Stress

Leaf disks from T0 transgenic and WT *N. tabacum* plants (8 weeks old) were floated on 0, 100, or 200 mM NaCl for 48 h under two different light intensities (150 or 450 μmol m^–2^ s^–1^) provided by cool white LEDs, with a photoperiod of 12 h. Salt stress was found to cause tissue damage. The extent of damage caused by the salt stress was able to be assessed by measuring the speed of leave pigments degradation. Therefore, chlorophylls and carotenoids were extracted and quantified from the leaf disks after the salt treatment.

**FIGURE 5:**
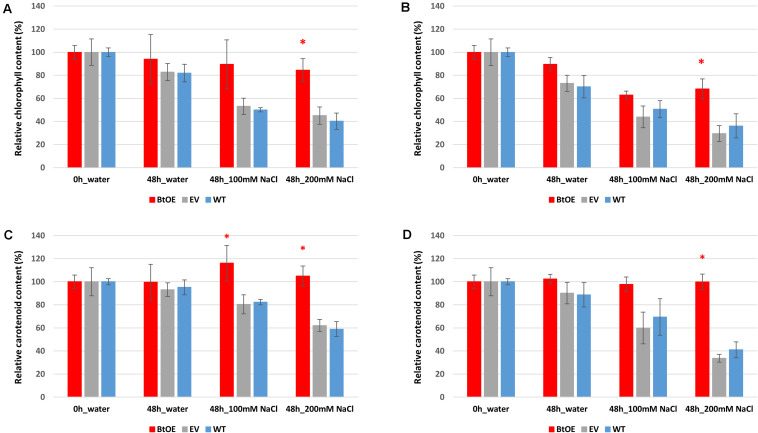
Effects of salt stress on pigment content of leaf disks. Relative total chlorophyll **(A,B)** and carotenoid **(C,D)** contents of *N. tabacum* leaf disks treated with 100 mM or 200 mM NaCl salt solutions under 150 μmol m^–2^ s^–1^ **(A,C)** and 450 μmol m^–2^ s^–1^ **(B,D)** light. Plant lines are wild type (WT), empty vector control (EV), and betacyanin overexpression transgenics (BtOE). Data are expressed as percentages of compound content in treated leaf disks versus that in untreated leaf disks. Means ± SE, *n* = 4. Asterisk indicates a statistically significant difference among three types of plants under same treatment (*P* < 0.05). The ANOVA analysis was performed independently on each treatment.

The total chlorophyll and carotenoid content was slightly higher in WT plants than BtOE plants under control conditions before treatment, with the EV plants intermediate between them (Supplementary Figure 5). This trend was reversed after the salt treatment, with WT and EV plants having significantly lower chlorophyll and carotenoid content than BtOE plants. The relative changes in photosynthetic pigment content are clearly seen when the data are displayed as relative content to that at the start of the salt treatment (Figures 5A,B). Under both light conditions, the total chlorophyll content decreased in WT, EV, and BtOE leaf disks under salt stress. However, in the BtOE leaf disks the chlorophyll content decreased more slowly than in WT and EV leaf disks, and after 48 h under high salt treatment, the relative chlorophyll content in BtOE leaf disks was significantly higher (30% and 20% higher, under high light or low light conditions, respectively) than in WT and EV leaf disks (*P* < 0.05) (Figures 5A,B). Carotenoid content decreased significantly in WT and EV leaf disks under all light intensities and salt treatments, while in BtOE leaf disks it did not change (Figures 5C,D). The relative carotenoid content in BtOE leaf disks was significantly higher (>19%) than in WT and EV leaf disks after 48 h high salt treatment under the low light condition (*P* < 0.05) (Figures 5C,D).

### Betacyanin Pigmentation Provided a Photo-Protection Benefit to Leaf Tissue Under Severe Salt Stress

**FIGURE 6:**
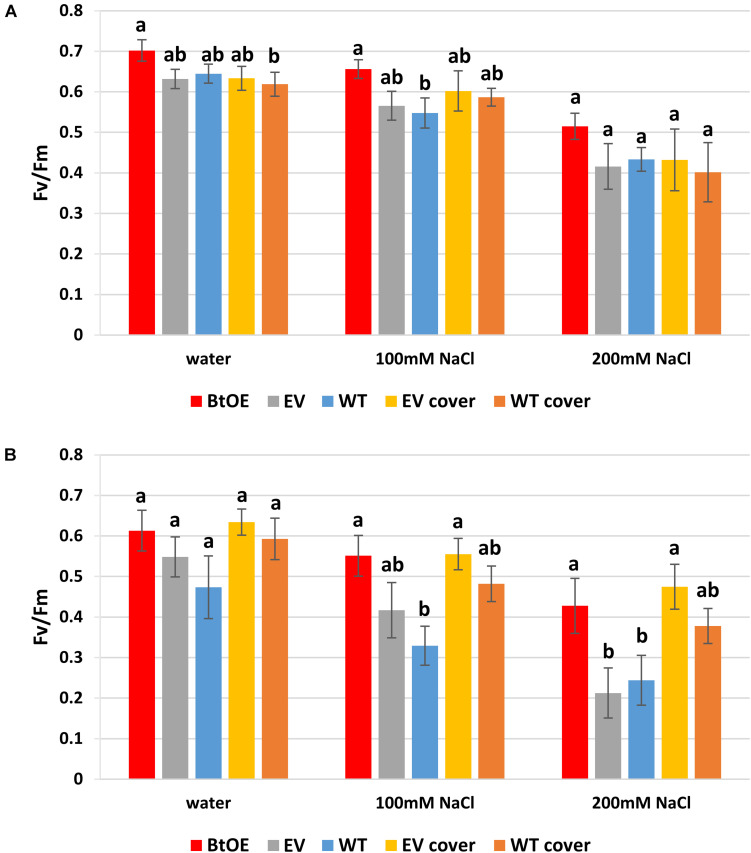
Effects of salt stress on the photochemical quantum yield of PSII (*F*_*v*_/*F*_*m*_) of *N. tabacum* leaf disks treated with water, 100 mM or 200 mM NaCl salt solutions under 150 μmol m^–2^ s^–1^ **(A)** or 450 μmol m^–2^ s^–1^ **(B)** light condition. Plant lines are wild type (WT), empty vector control (EV), and betacyanin overexpression transgenics (BtOE). Means ± SE, *n* = 4. Different letters indicate a statistically significant difference among three types of plant under same salt stress (*P* < 0.05). The ANOVA analysis was performed independently on each treatment.

To test if betacyanins can protect chloroplast function under salt stress, quantum yields of PSII (*F*_*v*_/*F*_*m*_) were measured on leaf disks treated with water or salt solutions. The *F*_*v*_/*F*_*m*_ of all three types of plants decreased after 48 h salt treatment (Figures 6A,B). The *F*_*v*_/*F*_*m*_ reduced less (about 7%) for the BtOE leaf disk than the WT and EV under salt stress and low light, but not to a statistically significant extent (Figure 6A). However, with the high light and high salt treatment, the *F*_*v*_/*F*_*m*_ was significantly lower in WT (18.2%) and EV (31.1%) than in the BtOE (*P* < 0.05) (Figure 6B).

The beneficial effect of betacyanin pigmentation on quantum yield of PSII during salt stress could be provided by light screening. To test this, a red polycarbonate filter with a similar absorption spectrum to betacyanins (530–550 nm) was applied on top of the container of EV and WT leaf disks to mimic the presence of the betacyanins. Covering with the red filter did not affect the EV and WT leaf disks’ *F*_*v*_/*F*_*m*_ under low light (Figure 6A). However, under the high light, especially with 200 mM salt stress, covering with the filter significantly enhanced the EV leaf disks’ *F*_*v*_/*F*_*m*_ by 36.2% (*P* < 0.05). Similarly, the application of the filter increased the *F*_*v*_/*F*_*m*_ of WT leaf disks under salt stress and high light conditions (by about 13%), although this was not statistically significant (Figure 6B). Together, the transgenic and light-filter results indicate the betacyanins provide photoprotection.

### Betacyanin Pigmentation Improved Photosynthetic Performance Under Salt Stress

**FIGURE 7:**
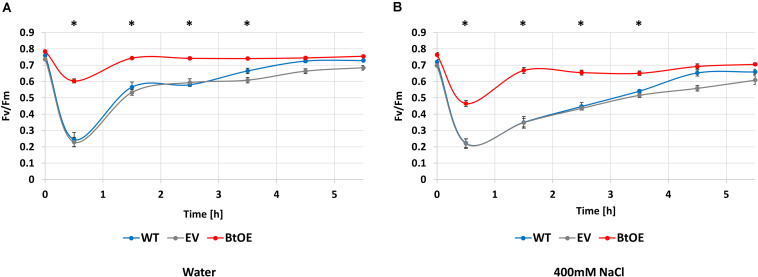
Betacyanins have a photoprotective effect in *N. tabacum* plants under salt stress. Changes in photochemical quantum yield of PSII (*F*_*v*_/*F*_*m*_) for wild type (WT), empty vector control (EV), and betacyanin overexpression (BtoE) *N. tabacum* plants after 2-week-old treatment with 40 mL of water **(A)** or 400 mM NaCl **(B)** daily. Plants were exposed to 1000 μmol m^–2^ s^–1^ white light for 30 min and then recovered for 5 h in darkness. Means ± SE, *n* = 4. Asterisks indicate a significant difference between BtOE and both WT and EV (one-way ANOVA) at each time point.

To assess if the salt tolerance enhancing effect of betacyanin production on photosynthetic ability observed in the leaf disk assay translated to the intact plants, the ability to recover from exposure to saturating light was assessed in salt-stressed WT, EV, and BtOE plants. *F*_*v*_/*F*_*m*_ measurements were taken before, during, and after exposure to 1000 μmol m^–2^ s^–1^ light (Figure 7). Before the salt stress treatment started, the initial *F*_*v*_/*F*_*m*_ of WT, EV, and BtOE plants was measured. All three genotypes had similar *F*_*v*_/*F*_*m*_ values around 0.8, confirming that the presence of betacyanin had no influence on *F*_*v*_/*F*_*m*_ under control, no stress conditions.

Photosynthesis recovery was assessed after 2 weeks of salt or control treatments. The decrease in *F*_*v*_/*F*_*m*_ after exposure to saturating light for 30 min was significantly higher in WT and EV than BtOE in both the water control (Figure 7A) and the plants treated with 400 mM NaCl (Figure 7B). In no salt stress plants, the *F*_*v*_/*F*_*m*_ decreased by 68% and 69% in WT and EV, respectively, while in BtOE the *F*_*v*_/*F*_*m*_ only decreased by 23%. In the salt treated plants exposure to saturating light reduced the *F*_*v*_/*F*_*m*_ by 69%, 68%, and 26% in WT, EV, and BtOE, respectively (*P* < 0.05). The betacyanic plants recovered significantly faster from photo-oxidative stress than WT and EV in both salt treated and control plants; it took 4 h for EV and WT plants to recover, while BtOE plants took only 1 h (Figures 7A,B). BtOE plants showed less reduction in the ratio of variable to maximum chlorophyll fluorescence after exposure to saturating light and a swifter recovery than WT and EV, suggesting that betacyanins had a photoprotective effect (Figures 7A,B), which is in consistent with the results of the leaf disk assay (Figure 6B).

### Betacyanin Pigmentation Increased Seedling Survival Under Severe Salt Stress

**FIGURE 8:**
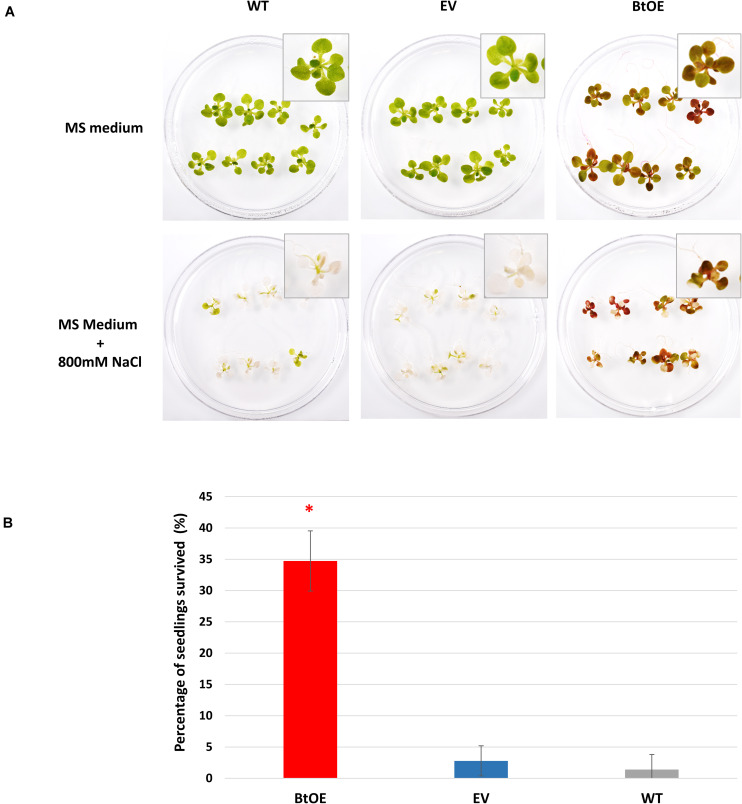
Betacyanin production increased *N. tabacum* seedling survival under severe salt stress. **(A)** photo of representative biological replicates of *N. tabacum* seedlings 8 days after being transferred onto normal growth (MS medium) or salt stress medium (MS with 800 mM NaCl). **(B)** percentage of seedlings that survived after severe salt stress treatment. Plant lines are wild type (WT), empty vector control (EV), and betacyanin overexpression transgenics (BtOE). Means ± SE, *n* = 3. Asterisks indicate significant difference relative to WT and EV (*P* < 0.05).

Survival is the biggest challenge for seedlings of glycophyte species under severe salt stress, especially when the stress is protracted. Thus, we assessed seedling survival rate of the WT, EV, and BtOE lines under high salt conditions. All three types grew equally well on control MS medium (Figure 8A). However, when stressed with 800 mM NaCl for 8 days, most of the WT and EV seedlings were achlorophyllous, while BtOE seedlings remained generally healthy, with just part of their leaves depigmented. After subsequent transfer to MS only medium for 2 weeks, about 35% of the BtOE seedlings recovered, while less than 5% of the EV or WT seedlings survived (*P* < 0.05) (Figure 8B).

### Betacyanin Production Increased Leaf Antioxidant Activity

**FIGURE 9:**
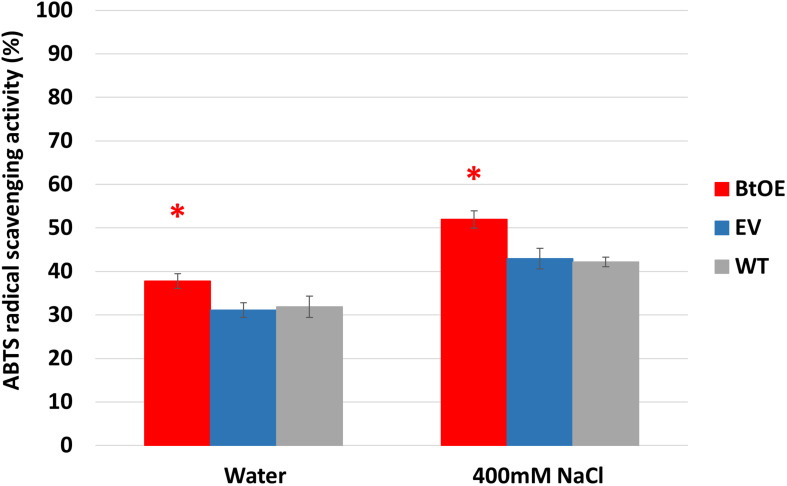
Betacyanin production enhanced overall antioxidant capability in leaves of *N. tabacum* grown under water or salt stress conditions. Plant lines are wild type (WT), empty vector control (EV), and betacyanin overexpression transgenics (BtOE). Means ± SE, *n* = 4. Asterisk indicates a statistically significant difference between BtOE and both EV and WT (*P* < 0.05).

To test if betacyanin production can deliver a free radical scavenging benefit under salt stress, total antioxidant capability was measured in leaf extracts of plants treated with water or salt stress. Leaf extracts of BtOE plants showed significant higher antioxidant activity than those of EV and WT under both control (about 5%) and salt stress (about 9%) conditions (*P* < 0.05). No significant difference was found between EV and WT leaf extract antioxidant activity (Figure 9).

To examine whether the increased antioxidant activity could be due to an indirect effect, i.e., betacyanin production inducing gene expression in endogenous pathways, we measured transcript abundance of three key antioxidant-related genes, peroxidase (POX), superoxide dismutase (SOD), and catalase (CAT) (Supplementary Table 1, Supplementary Methods). We also included in the analysis one salt tolerance related gene, osmotin, which is involved in osmotic adjustments. No significant difference was observed among BtOE, EV, and WT plants in expression of any of the genes under either water or salt stressed conditions (Supplementary Figure 6). Thus, the increased antioxidant activity is likely directly due to the betacyanins.

## Discussion

Introducing three betalain biosynthetic genes, *BvCYP76AD1, MjcDOPA5GT*, and *BvDODA1*, resulted in heterologous betalain production in *N. tabacum*. These three betalain genes have been previously used to introduce betalain production into *N. tabacum* and other solanaceous species (anthocyanic rather than betalainic plants) as well as microorganisms (Polturak et al., 2016, 2017). The red leaf pigments in our BtOE plants were confirmed as betacyanins.

When plants are expose to salt stress, their photosynthetic activity is significantly depressed, which leads to problems with excessive light energy (Takahashi and Murata, 2008; van Zelm et al., 2020). The excess excitation energy within the photosynthetic apparatus can cause impairment of chloroplast performance, revealed by a rapid decline in *F*_*v*_/*F*_*m*_. Therefore, light screening may provide a significant benefit to plants undergoing salt stress under relatively high light conditions, and this is supported by the results for our betacyanic *N. tabacum*. The BtOE lines had improved *F*_*v*_/*F*_*m*_ values under stress conditions compared with control lines, and recovered significantly faster (Figure 7). Notably, betacyanin pigment was abundant throughout leaf tissue (Figure 3B), and so could reduce the photosynthetically active light passing to photosynthetic cells beneath (Li et al., 2019). Covering WT and EV leaf disks with a red filter simulating betacyanins had a similar effect on *F*_*v*_/*F*_*m*_ as *in planta* betacyanin production (Figure 6), supporting light screening as one of the principal benefits of betacyanin production. Betacyanins have similar spectral properties as anthocyanins (Neill and Gould, 1999; Li et al., 2019), and there is abundant evidence that anthocyanins assist in protecting chloroplasts from potentially damaging effects of supernumerary photons directly by abating incident quantum fluxes (Landi et al., 2015).

The photoprotective function of betacyanins has also been reported in studies on betalainic species. In *Amaranthus cruentus*, photoinhibition was greater in green leaves than in betacyanic leaves and it was suggested that betacyanins reduced the excitation pressure on PSII by attenuating the harmful excess light (Nakashima et al., 2011). *Suaeda salsa* seedlings that accumulated betacyanins not only showed a higher resistance to photoinhibition but also recovered more quickly after light stress (Wang and Liu, 2007). In *D. australe*, photoprotection was indicated as part of the mechanism by which betacyanins increased salt stress tolerance, as pigmented plants had greater PSII quantum yields and photochemical quenching ability and recovered swifter after salt treatment than the green plants (Jain and Gould, 2015; Jain et al., 2015). Moreover, betacyanic *D. australe* leaves absorbed more green and yellow light (Jain and Gould, 2015). A photoprotective role for betacyanins could explain their relative abundance in species found in stressful environments such as salt marshes and deserts (Ehrendorfer, 1976; Jain and Gould, 2015).

In the presence of abiotic stresses, such as drought or salinity, seedling establishment is a critical process during the life cycle of a plant (Bohnert et al., 1995; Adolf et al., 2013). At this life stage, halophytes have a particular advantage over glycophytes on saline soils (Malcolm et al., 2003; Debez et al., 2004; van Zelm et al., 2020). BtOE seedlings had a greater survival rate after exposure to high salinity (Figure 8B). Similar to as has been observed for seeds of some naturally betalanic plants, such as *Chenopodium quinoa* (Escribano et al., 2017), the BtOE seeds contain betacyanins (Figures 2A–C), and leaves, stem and roots of the germinated seeds had a strong red-violet hue (Figures 2D,E). The higher survival rate of BtOE seedling than EV and WT (Figure 8B) could be because of the photoprotection benefit delivered by the presence of betacyanins in leaves and/or increased ROS scavenging capacity throughout the seedling. Increased production of the more common vacuolar red pigments of plants, anthocyanins, has also been shown to improve abiotic stress tolerance. For example, overproduction of anthocyanin significantly enhanced Arabidopsis seedling survival under salt stress, while reduction of anthocyanin production decreased survival rates (Oh et al., 2011).

In addition to photoprotection, the production of betacyanins in *N. tabacum* may also have contributed to improved salt stress tolerance through increased capacity for ROS scavenging, as the ABTS assay showed that BtOE lines had significantly increased total antioxidant capacity under either control or stress conditions (Figure 9). The ABTS assay has been showed to give a good measure of antioxidant capacity in *N. tabacum*, with both the ABTS and 2,2-diphenyl-1-picrylhydrazyl (DPPH) assays giving very similar results (Naing et al., 2017, 2018). Both light and salt stress can induce the formation of supernumerary ROS (van Zelm et al., 2020), such as singlet oxygen and H_2_O_2_, which can cause oxidative damage to cell membranes, proteins, and DNA (Gill and Tuteja, 2010). Both betanin and isobetanin, two of the three types of betacyanin identified in BtOE leaves, have been shown to have ROS scavenging properties *in vitro* that are approximately three times greater than an equivalent amount of ascorbic acid (Cai et al., 2005). Also, purified betacyanin from *Suaeda japonica* could prevent H_2_O_2_-induced protein oxidation (Hayakawa and Agarie, 2010). Similar to the BtOE results for salt stress, transgenic betacyanic tomato (*Solanum lycopersicum*) plants had increased antioxidant capacity (60% increase was measured in a fruit extract); and increased ROS scavenging was suggested as contributing to the enhanced resistance against phytopathogenic *Botrytis cinerea* in transgenic betacyanic *N. tabacum* (Polturak et al., 2017). An antioxidant benefit associated with betacyanins has also been suggested for naturally betacyanic species. For example, betacyanin production is induced by H_2_O_2_ application to *S. salsa* roots (Wang and Liu, 2007) or *Berberis vulgaris* leaves (Sepúlveda-Jiménez et al., 2004). As with light screening, anthocyanins as well as betacyanins have been shown to have ROS scavenging activity. In an *in vivo* study, anthocyanins were identified as one of the major low molecular weight antioxidants in leaves, significantly enhanced H_2_O_2_ scavenging (Gould et al., 2002). Transgenic *N. tabacum* and *Arabidopsis thaliana* plants with increased anthocyanin production had higher total ROS scavenging activity than control plants (Li et al., 2017; Naing et al., 2017, 2018).

The relative benefits from anthocyanins of direct light screening compared with ROS scavenging is of much debate (Agati et al., 2012, 2013, 2020), and the same arguments can be applied to betalains (Davies et al., 2018). A common observation, supporting the proposition that they principally have a screening function, is that betacyanins are predominantly located in epidermal cells [e.g., in *D. australe* (Jain et al., 2015)], while ROS generation is primarily in sub-epidermal photosynthetic palisade cells. However, for BtOE *N. tabacum*, the promoters of the transgenes allowed less restricted pigmentation, with betacyanin accumulating throughout the leaf including palisade and spongy mesophyll cells (Figure 3B). Thus, betacyanins could have provided an extra benefit in this case even if they do not normally have this role. In addition to epidermal-localization, another argument that could be raised against betacyanins functioning as key antioxidants is that they are localized within the vacuole while principal sources of ROS are chloroplasts, mitochondria and peroxisomes (Apel and Hirt, 2004). Although the full sub-cellular localization of betalains has not been confirmed for betalain over-production transgenics, they appeared to be accumulated throughout the vacuole at least (Figures 3E,F). However, there is evidence that H_2_O_2_ can diffuse easily between cellular compartments and so can come into contact with the secondary metabolites in the vacuole (Gould et al., 2002; Agati et al., 2012). Moreover, detailed arguments for an effective role of flavonoids as antioxidants and/or activity within the REDOX system have been recently presented (Chapman et al., 2019; Agati et al., 2020; Gayomba and Muday, 2020), and many of these proposals could also apply to betacyanins. An ability to reduce H_2_O_2_ concentrations is also supported by the finding that in *Catharanthus roseus* 90% of total leaf class III peroxidase (POX) activity was localized in the vacuoles (Ferreres et al., 2011), and the vacuolar-located POX and secondary metabolites (SMs) were proposed to have a key role in the homeostasis of H_2_O_2_ content (Ferreres et al., 2011). An indirect observation supporting a ROS scavenging role by betacyanins in the BtOE *N. tabacum* is the increased carotenoid content, compared with WT or EV lines, under both salt and light stress (Figure 5). Carotenoids are thought to be important ROS scavengers that are degraded during the reaction, especially in association with singlet oxygen scavenging during photo-oxidative stress (Burke et al., 2000; Ramel et al., 2012; Foyer, 2018). Therefore, the increased carotenoid content may reflect reduced ROS amounts. However, it is difficult to separate the possible ROS scavenging role from a reduction in ROS resulting from reduced photo-oxidative stress following direct light screening by the betacyanins.

The WT (but not EV) plants had a small, but statistically significant, higher amount of carotenoids and chlorophyll under non-stress conditions. Previous studies have found the similar trends. For example, betacyanins were negatively correlated with chlorophyll and carotenoids content in shoots of *Salicornia brachiate* (Parida et al., 2018). The same trend has also been reported for anthocyanin and chlorophyll content (e.g., Ren et al., 2019), suggesting the increased light-screening can lower absolute amounts of photosynthetic pigments under non-stress conditions.

Scavenging of ROS depends on a range of enzymatic components activity within the cell (Das and Roychoudhury, 2014). Transgenic *N. tabacum* plants with increased anthocyanin production (through overexpression of two anthocyanin-related transcription factors) also had increased transcript abundance for several important antioxidant enzymes and a salt tolerance-related genes such as POX, SOD, CAT, and osmotin (Naing et al., 2017, 2018). However, we found that introduction of betacyanin biosynthesis into *N. tabacum* using biosynthetic genes did not cause increased transcript abundance for POX, SOD, CAT, or osmotin (Supplementary Figure 6), nor changed phenylpropanoids profile (Supplementary Figure 4). It is possible that the transcription factor transgenes used for increasing anthocyanin biosynthesis also activate other stress-related pathways, and this does not occur with the specific betalain biosynthetic transgenes used. Further analysis on oxidative damage parameters and cell membrane function in the future study will be needed in order to elucidate the antioxidant effect of betacyanins in plant *in vivo*.

Anthocyanins and betacyanins have similar light screening properties. If light screening is the only mechanism by which salinity tolerance is enhanced by these pigments, then betacyanins may not offer better salt-tolerance properties than anthocyanins; yet they are commonly produced in halophytes such as *D. australe*. It could be that betacyanins contribute to salinity tolerance when produced in *D. australe* in additional ways that are not transferred across to *N. tabacum*. Knowledge is scant on many aspects of betalain production in comparison to our understanding for anthocyanins. Generally, little is known about betalain intra- or inter-cellular transport and accumulation mechanisms, whether other compounds are produced from the intermediates within the betalain biosynthetic pathway, or if transcriptional regulators of the pathway also regulate other metabolic responses. Any one of these could also contribute to improved salinity responses, and highlights the need for further research on the betalain pathway. The evolution of betalain pigmentation and loss of anthocyanin pigmentation in the Caryophyllales is intriguing. The ability to make transgenic plants of a single species that accumulate either type of red pigment, or both together, should aid studies to elucidate their comparative advantages under different stresses.

In addition to photoprotection and/or ROS scavenging, betacyanins could potentially contribute to salt tolerance by reducing uptake of Na ions or causing the Na ions to be sequestered away from sensitive cellular components. The activity of an H^+^-ATPase (V-ATPase) involved in Na^+^ vacuolar transport increased more in red betacyanic leaves than green leaves of *Suaeda salsa* when plants were exposed to salt stress (Wang and Liu, 2007), suggesting betacyanins could alter Na^+^ sequestration. However, we found no difference in either Na^+^ concentration (Supplementary Figure 7, Supplementary Methods) or distribution (Supplementary Figure 8, Supplementary Methods) between leaves of WT or BtOE plants with or without 400 mM NaCl treatment. Thus, in the transgenic *N. tabacum*, light screening and/or ROS scavenging are the more probable mechanism for conferring salt stress tolerance. It is possible that in the genetic background of halophytes such as *Suaeda salsa* and *Disphyma australe* there are additional components that act with the betacyanins to alter Na^+^ transport.

In conclusion, we demonstrated a causative role for betacyanins in salt tolerance and showed it is possible to transfer advantages of betacyanin pigmentation to a non-betalainic species. The betacyanins provided photoprotective light screening and potentially functioned as ROS-scavengers. Finally, as soil salinity is one of the most important agricultural problems, recently estimated to cause US$ 27.3 billion in global agricultural losses (Qadir et al., 2014), introducing betacyanin biosynthesis could be a new approach to increase the salt tolerance of crops for production on salinizing soils.

## Data Availability Statement

The original contributions presented in the study are included in the article/Supplementary Material, further inquiries can be directed to the corresponding author.

## Author Contributions

KG, KS, KD, and KR conceived the project. YZ made the transgenics and conducted the experiments. TK and YZ performed photosynthesis measurement and sodium ion concentration and distribution analysis. DL, SA, and TM conducted metabolite analyses. YZ, KS, and KD contributed to the experimental design, interpretation of the data, and wrote the manuscript. KG and KR contributed to the manuscript editing. All authors contributed to the article and approved the submitted version.

## Conflict of Interest

The authors declare that the research was conducted in the absence of any commercial or financial relationships that could be construed as a potential conflict of interest.

## Footnotes

1 http://www.genscript.com

2 http://www.walz.com/downloads/manuals/pam-2500/PAM_2500_07-2.pdf

## Supplementary Material

Supplementary Figure 1 The betalain biosynthesis pathway. Simplified representation of the betalain biosynthetic pathway leading to the production of betacyanins and betaxanthins. Enzymes shown are the cytochrome P450 enzymes CYP76AD1 and CYP76AD6, DOPA 4, 5-dioxygenase (DODA), *cyclo*-DOPA 5-*O*-glucosyltransferase (cDOPA 5GT), betanidin 5-*O*-glucosyltransferase (Betanidin 5GT), and betanidin 6-*O*-glucosyltransferase (Betanidin 6GT). The condensation reactions of betalamic acid with amino acids/amines to form betacyanin and betaxanthin pigments occur spontaneously.

Supplementary Figure 2 Agarose gel photo of representative RT-PCR analysis of gene expression in leaf tissue of a representative transgenic *N. tabacum* line. WT, wild type; EV, pART27 empty vector control; BtOE, betacyanin over expressing transgenic line; Positive, betalain binary plasmid; Negative, DNA free water. CYP76AD1, *B. vulgaris* cytochrome P450 (HQ656023.1); cDOPA5GT, *Mirabilis jalapa* cyclo-DOPA-5-*O*-glucosyltransferase (AB182643.1); DODA1, *B. vulgaris* DOPA 4,5-dioxygenase (HQ656027.1); nptII, kanamycin resistance marker.

Supplementary Figure 3 Identification of betaxanthins in *N. tabacum*. HPLC chromatogram of *N. tabacum* leaf extract. The horizontal axis indicates the retention time (min), whereas the vertical axis indicates the signal intensity (l V). 1, 2, and 3 are bleed over peaks of three betacyanins (showed in Figure 4). The peaks of three types of betaxanthins are indicated with asterisk.

Supplementary Figure 4 Phenylpropanoids profile in *N. tabacum*. HPLC chromatogram of *N. tabacum* leaf extract at **(A)** 280 nm and **(B)** 340 nm. The horizontal axis indicates the retention time (min), whereas the vertical axis indicates the signal intensity (l V).

Supplementary Figure 5 Effects of salt stress on pigment content of leaf disks. Absolute total chlorophyll **(A,B)** and carotenoid **(C,D)** contents of *N. tabacum* leaf disks treated with 100 mM or 200 mM NaCl salt solutions under 150 μmol m^–2^ s^–1^ **(A,C)** and 450 μmol m^–2^ s^–1^ **(B,D)** light. Plant lines are wild type (WT), empty vector control (EV), and betacyanin overexpression transgenics (BtOE). Data is expressed as percentages of compound content in treated leaf disks versus that in untreated leaf disks. Means ± SE, *n* = 4. Different letters indicate a statistically significant difference among three types of plants under the same treatment (*P* < 0.05). The ANOVA analysis was performed independently on each treatment.

Supplementary Figure 6 qRT-PCR analysis of antioxidant-related gene expression of *N. tabacum* plants. **(A)** POX, **(B)** SOD, **(C)** CAT, and **(D)** Osmotin. *N.* tabacum elongation factor 1-alpha was used as reference gene. Means ± SE, *n* = 4.

Supplementary Figure 7 Betacyanins do not affect sodium accumulation in *N. tabacum* plants under salt stress. Changes in total Na^+^ content for wild type (WT) and betacyanin overexpression (BtOE) *N. tabacum* plants after 2 weeks treatment with 50 mL of water or 400 mM NaCl daily. Means ± SE, *n* = 4.

Supplementary Figure 8 Betacyanins do not affect sodium distribution in leaves of *N. tabacum* plants under salt stress. Changes in Na^+^ distribution in leaves of wild type (WT) and betacyanin overexpression (BtOE) *N. tabacum* plants after 2 weeks treatment with 50 mL of water or 400 mM NaCl daily. Green fluorescence indicates cytosolic Na^+^ using sodium green tetraacetate.
