# Supplementary material for: Production of Betacyanins in Transgenic Nicotiana tabacum Increases Tolerance to Salinity
Source: Front Plant Sci. 2021 Apr 30;12:653147. doi: 10.3389/fpls.2021.653147 (PMC8121086; doi:10.3389/fpls.2021.653147)
Supplement: Supplementary Figure 1 — The betalain biosynthesis pathway. Simplified representation of the betalain biosynthetic pathway leading to the production of betacyanins and betaxanthins. Enzymes shown are the cytochrome P450 enzymes CYP76AD1 and CYP76AD6, DOPA 4, 5-dioxygenase (DODA), cyclo-DOPA 5-O-glucosyltransferase (cDOPA 5GT), betanidin 5-O-glucosyltransferase (Betanidin 5GT), and betanidin 6-O-glucosyltransferase (Betanidin 6GT). The condensation reactions of betalamic acid with amino acids/amines to form betacyanin and betaxanthin pigments occur spontaneously. [file Data_Sheet_1.zip › Supplementary Methods .DOCX]

**Supplementary methods**

**RNA extraction and PCR analysis**

Total RNA was isolated from frozen ground plant leaves by the CTAB/chloroform extraction method described in Gambino *et al.* (2008). RNA treated with 1 µg of DNaseI (Roche Diagnostics, Mannheim, Germany) was used to make first strand cDNA using SuperScript™ II Reverse Transcriptase (Invitrogen, California, USA). The Polymerase chain reaction (PCR) analysis for transgene transcript detection was carried out in 50 µl PCR reaction mixtures with 1X PCR buffer, 0.2 mM dNTPs, 1.5 mM MgCl_2_, 1 U Platinum^®^ Taq DNA Polymerase (Invitrogen), 0.2 µM of each primer, and 1 µl of 1000X diluted cDNA, using a Bio-Rad PCR system (T100™ Thermal Cycler, Bio-Rad Laboratories, California, USA). The qPCR reactions were carried out using a LightCycler® 480 SYBR Green I Master kit (Roche Diagnostics) and performed with a LightCycler® 480 Instrument II (384-well; Roche Diagnostics) according to the manufacturer’s instructions. The Cp value was calculated using the algorithm of “Abs Quant / 2nd Derivative Max” present in LightCycler® 480 Software (version 1.5). Data were normalized to the reference gene (*N. tabacum* *elongation factor 1-alpha*) and relative transcriptional changes were calculated using the ΔΔCt method (Dvinge & Bertone, 2009). Primer sequences and annealing temperature are given in Table S1.

**Sodium ion content measurement**

T2 transgenic and WT plants were grown from seeds in pots (85 mm × 85 mm × 100 mm) in the greenhouse as described above, for 2 months. Four independent lines of each type of transgenic plant were used. Plants were irrigated daily with 50 ml of tap water or 400 mM NaCl for 2 weeks. The third mature leave of each plant was used to measure total Na^+^ content before and after salt treatment. Leaves were freeze dried and ground into fine powder, and 50 mg of each sample was used for Na^+^ extraction. Samples were digested in 10% HNO_3_ and 5.2% HCl at room temperature for 30 min and then diluted with distilled water to 1.4% HNO_3_ and 0.74% HCl. A calibration curve for Na^+^ measurement was made with 0, 1.25, 2.5, 3.75, 5, 6.25, 7.5, 8.75 and 10 μg/ml NaCl in distilled water with 1.4% HNO_3_ and 0.74% HCl. The Na^+^ content was determined with a ICE3000 Series AAspectrometer (ThermoScientific, Waltham, Massachusetts, USA) combined with an ASX-520 Autosampler (CETAC Technologies, Omaha, Nebraska, USA).

**Sodium ion distribution analysis**

T1 transgenic and WT plants were grown from seeds in pots (85 mm × 85 mm × 100 mm) in the greenhouse as decribed above, for 2 months. Four independent lines of each type of transgenic plant were used. Plants were irrigated daily with 50 ml of tap water or 400 mM NaCl for 2 weeks. The third mature leave of each plant was used to examine the Na^+^ distribution with or without salt treatment.

The distribution of Na^+^ in control and salt treated leaves was examined by fluorescence microscopy using the Sodium Green fluorophore (Hamaji *et al.*, 2009). A small piece of collected leaves tissue was transferred into an Eppendorf tube containing 1 ml of 10 μM Sodium Green tetraacetate (Thermofisher Scientific, Waltham, Massachusetts, USA) in dimethyl sulfoxide (Sigma-Aldrich, St. Louis, Missouri, USA) and held in the dark at room temperature for 3h. Thin transverse-sections were hand-cut and examined by epifluorescence in an Olympus AX70 compound microscope (Olympus Optical Co., Hamburg, Germany) using a 460-490 nm excitation waveband and collecting emission between 515 nm and 550 nm.

**References:**

Dvinge H, Bertone P. 2009. HTqPCR: high-throughput analysis and visualization of quantitative real-time PCR data in R. *Bioinform* 25: 3325-3326. doi: 10.1093/bioinformatics/btp578

Gambino G, Perrone I, Gribaudo I. 2008. A rapid and effective method for RNA extraction from different tissues of grapevine and other woody plants. *Phytochem Anal* 19: 520-525. doi: 10.1002/pca.1078

Hamaji K, Nagira M, Yoshida K, Ohnishi M, Oda Y, Uemura T, Goh T, Sato MH, Morita MT, Tasaka M, *et al.* 2009. Dynamic aspects of ion accumulation by vesicle traffic under salt stress in Arabidopsis. *Plant Cell Physiol* 50: 2023–2033. doi:10.1093/pcp/pcp143
