# Supplementary figures and images for: Production of Betacyanins in Transgenic Nicotiana tabacum Increases Tolerance to Salinity
Source: Front Plant Sci. 2021 Apr 30;12:653147. doi: 10.3389/fpls.2021.653147 (PMC8121086; doi:10.3389/fpls.2021.653147)

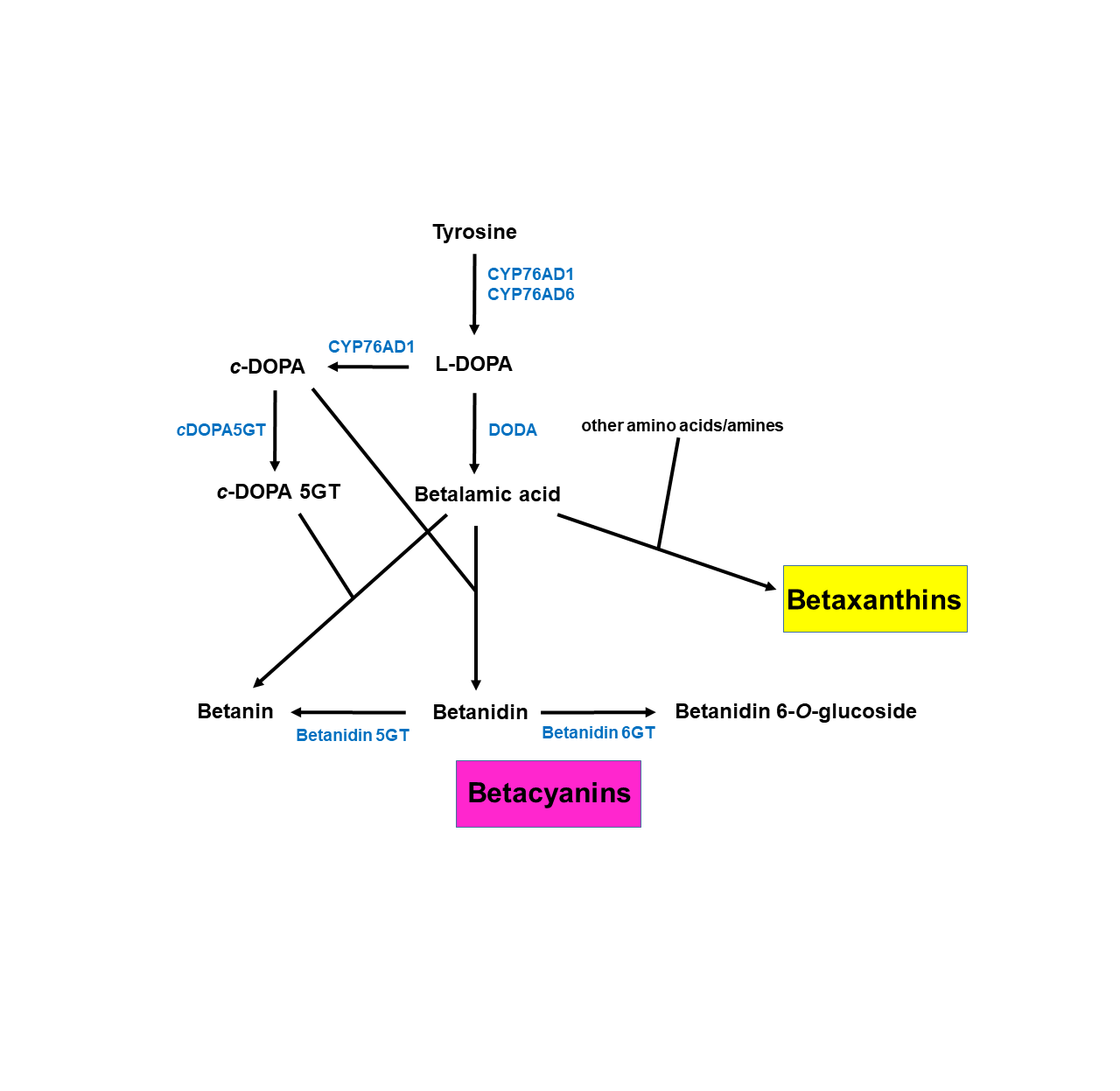

Supplement: Supplementary Figure 1 — The betalain biosynthesis pathway. Simplified representation of the betalain biosynthetic pathway leading to the production of betacyanins and betaxanthins. Enzymes shown are the cytochrome P450 enzymes CYP76AD1 and CYP76AD6, DOPA 4, 5-dioxygenase (DODA), cyclo-DOPA 5-O-glucosyltransferase (cDOPA 5GT), betanidin 5-O-glucosyltransferase (Betanidin 5GT), and betanidin 6-O-glucosyltransferase (Betanidin 6GT). The condensation reactions of betalamic acid with amino acids/amines to form betacyanin and betaxanthin pigments occur spontaneously. [file Data_Sheet_1.zip › Supplementary Figure 1.TIF]

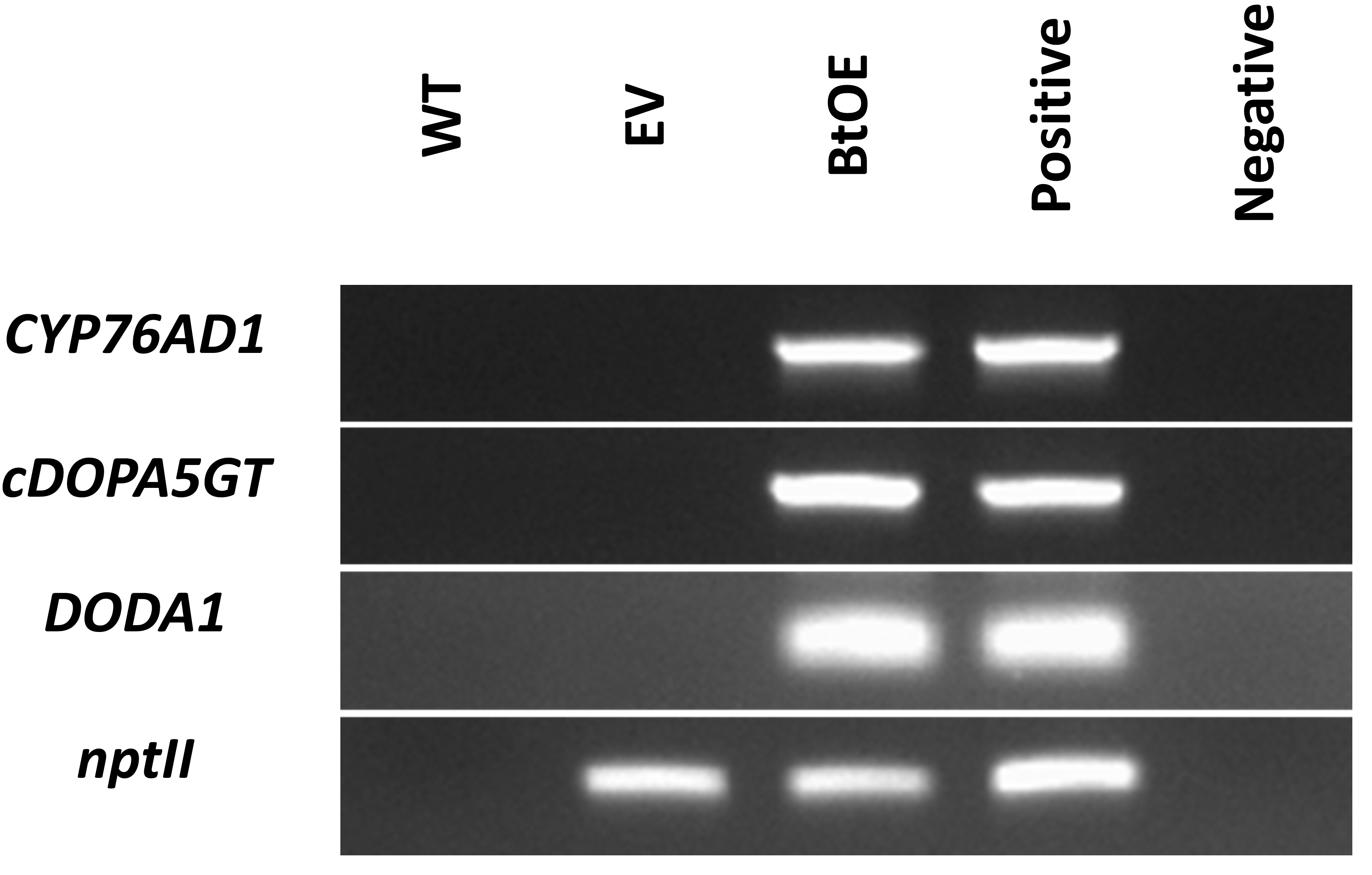

Supplement: Supplementary Figure 1 — The betalain biosynthesis pathway. Simplified representation of the betalain biosynthetic pathway leading to the production of betacyanins and betaxanthins. Enzymes shown are the cytochrome P450 enzymes CYP76AD1 and CYP76AD6, DOPA 4, 5-dioxygenase (DODA), cyclo-DOPA 5-O-glucosyltransferase (cDOPA 5GT), betanidin 5-O-glucosyltransferase (Betanidin 5GT), and betanidin 6-O-glucosyltransferase (Betanidin 6GT). The condensation reactions of betalamic acid with amino acids/amines to form betacyanin and betaxanthin pigments occur spontaneously. [file Data_Sheet_1.zip › Supplementary Figure 2.TIF]

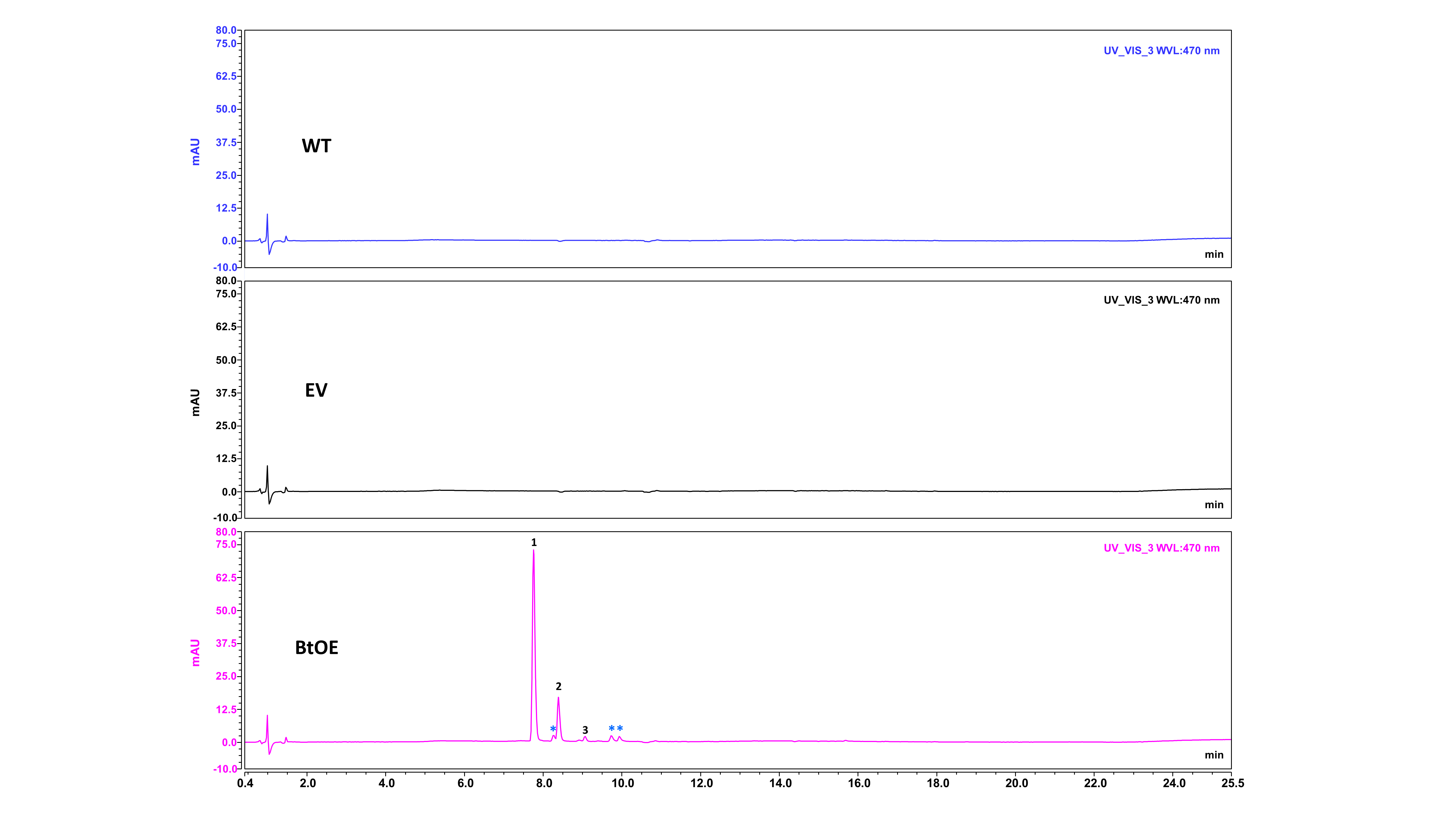

Supplement: Supplementary Figure 1 — The betalain biosynthesis pathway. Simplified representation of the betalain biosynthetic pathway leading to the production of betacyanins and betaxanthins. Enzymes shown are the cytochrome P450 enzymes CYP76AD1 and CYP76AD6, DOPA 4, 5-dioxygenase (DODA), cyclo-DOPA 5-O-glucosyltransferase (cDOPA 5GT), betanidin 5-O-glucosyltransferase (Betanidin 5GT), and betanidin 6-O-glucosyltransferase (Betanidin 6GT). The condensation reactions of betalamic acid with amino acids/amines to form betacyanin and betaxanthin pigments occur spontaneously. [file Data_Sheet_1.zip › Supplementary Figure 3.TIF]

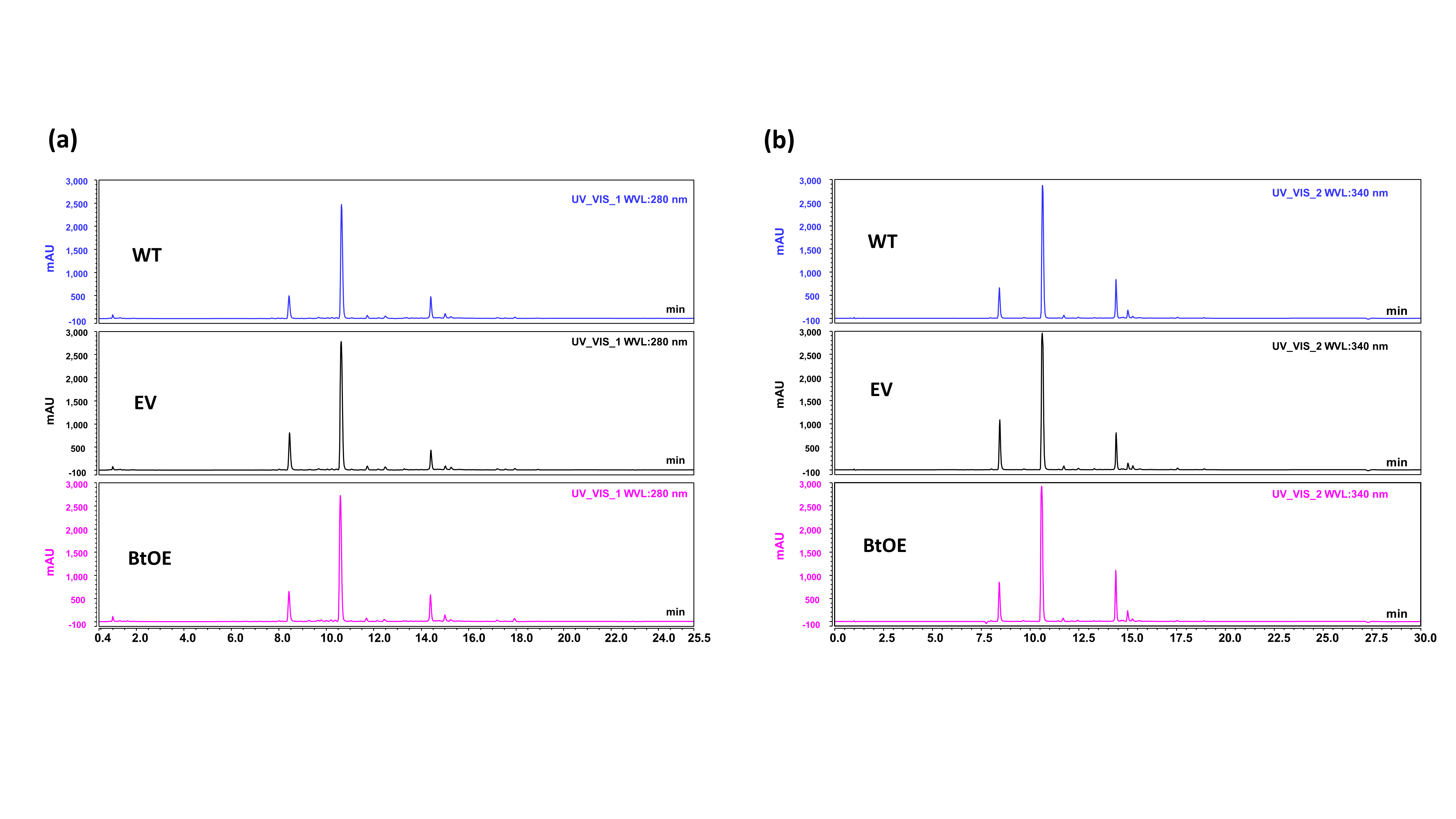

Supplement: Supplementary Figure 1 — The betalain biosynthesis pathway. Simplified representation of the betalain biosynthetic pathway leading to the production of betacyanins and betaxanthins. Enzymes shown are the cytochrome P450 enzymes CYP76AD1 and CYP76AD6, DOPA 4, 5-dioxygenase (DODA), cyclo-DOPA 5-O-glucosyltransferase (cDOPA 5GT), betanidin 5-O-glucosyltransferase (Betanidin 5GT), and betanidin 6-O-glucosyltransferase (Betanidin 6GT). The condensation reactions of betalamic acid with amino acids/amines to form betacyanin and betaxanthin pigments occur spontaneously. [file Data_Sheet_1.zip › Supplementary Figure 4.TIF]

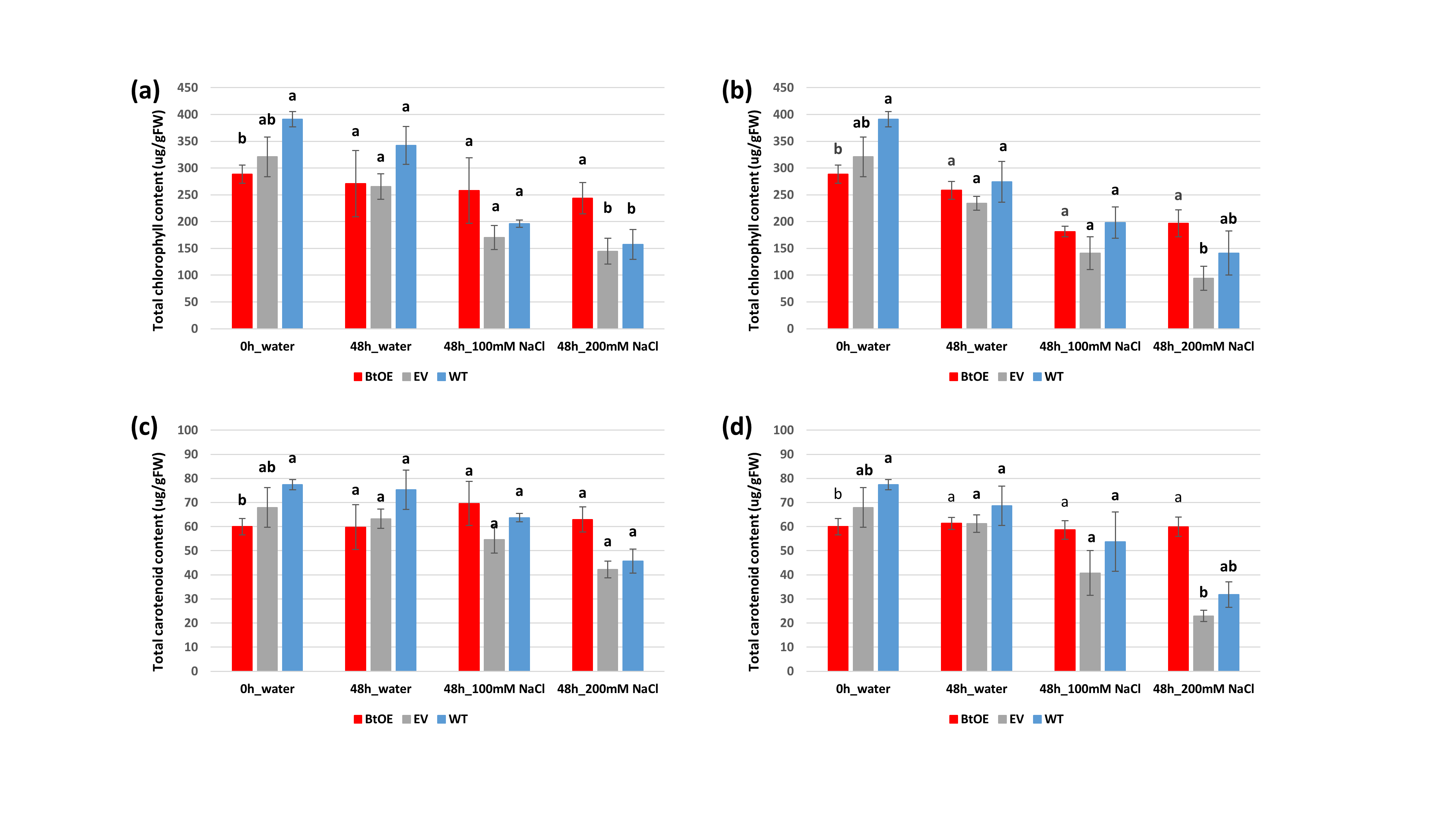

Supplement: Supplementary Figure 1 — The betalain biosynthesis pathway. Simplified representation of the betalain biosynthetic pathway leading to the production of betacyanins and betaxanthins. Enzymes shown are the cytochrome P450 enzymes CYP76AD1 and CYP76AD6, DOPA 4, 5-dioxygenase (DODA), cyclo-DOPA 5-O-glucosyltransferase (cDOPA 5GT), betanidin 5-O-glucosyltransferase (Betanidin 5GT), and betanidin 6-O-glucosyltransferase (Betanidin 6GT). The condensation reactions of betalamic acid with amino acids/amines to form betacyanin and betaxanthin pigments occur spontaneously. [file Data_Sheet_1.zip › Supplementary Figure 5.TIF]

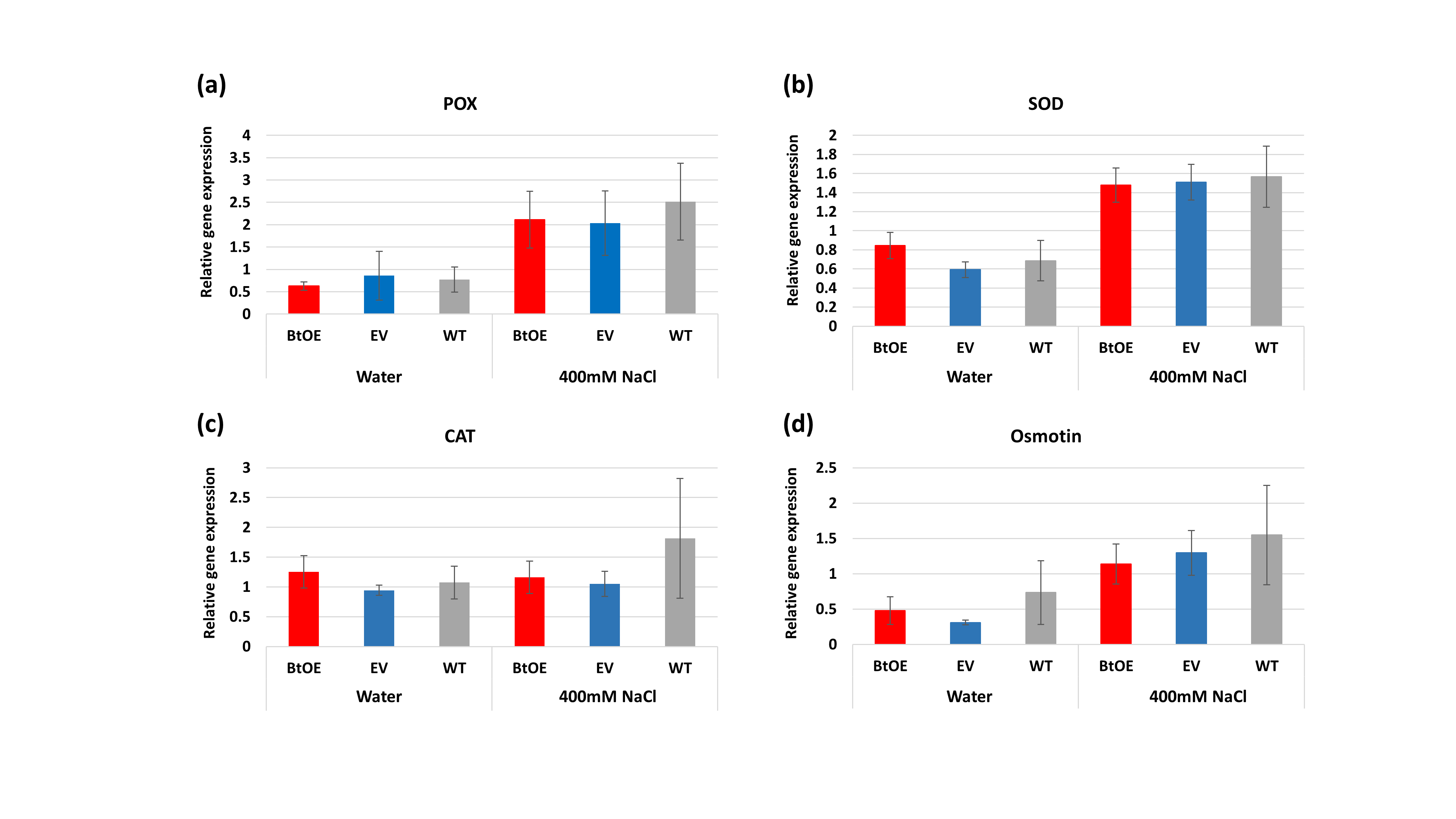

Supplement: Supplementary Figure 1 — The betalain biosynthesis pathway. Simplified representation of the betalain biosynthetic pathway leading to the production of betacyanins and betaxanthins. Enzymes shown are the cytochrome P450 enzymes CYP76AD1 and CYP76AD6, DOPA 4, 5-dioxygenase (DODA), cyclo-DOPA 5-O-glucosyltransferase (cDOPA 5GT), betanidin 5-O-glucosyltransferase (Betanidin 5GT), and betanidin 6-O-glucosyltransferase (Betanidin 6GT). The condensation reactions of betalamic acid with amino acids/amines to form betacyanin and betaxanthin pigments occur spontaneously. [file Data_Sheet_1.zip › Supplementary Figure 6.TIF]

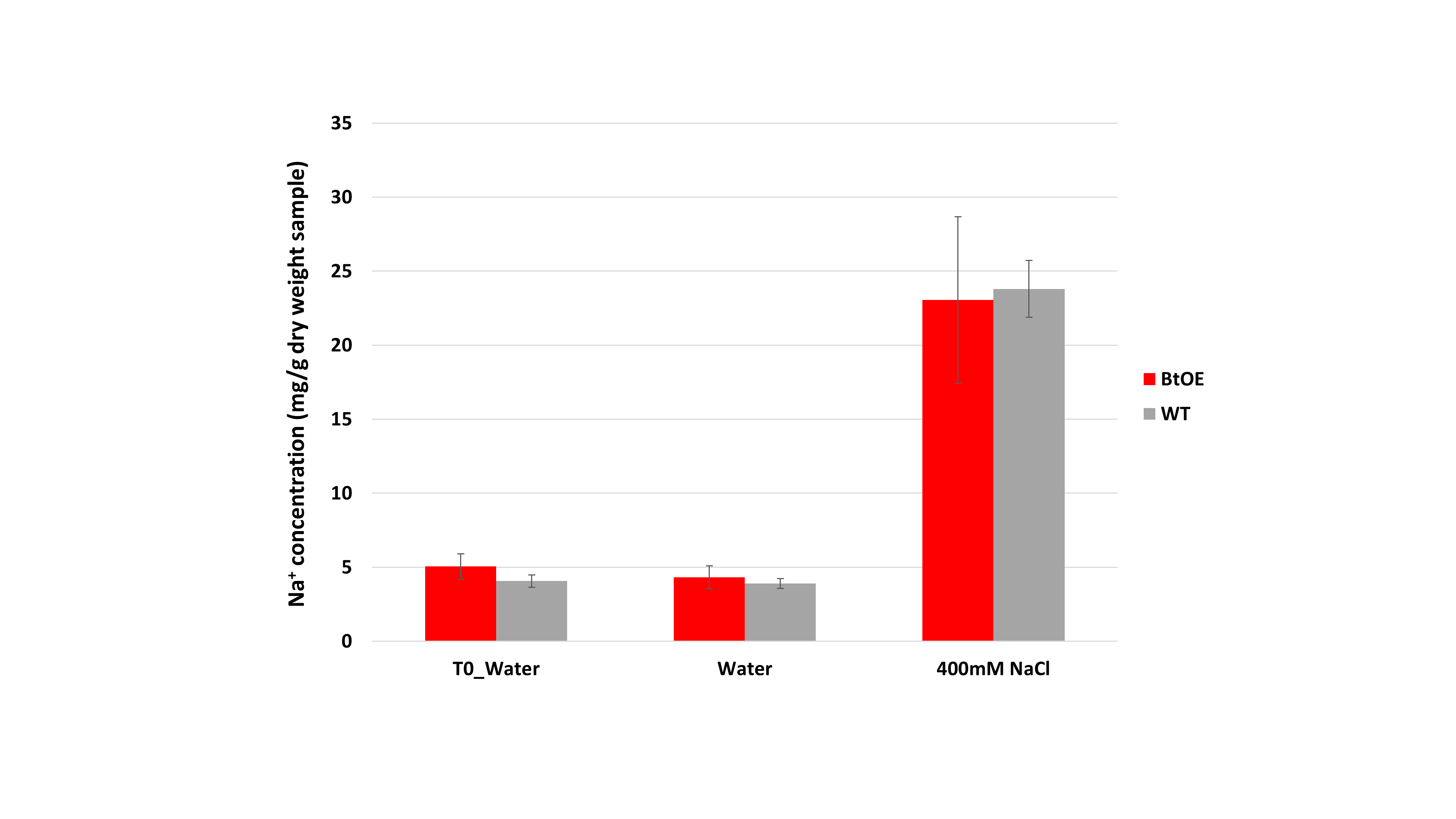

Supplement: Supplementary Figure 1 — The betalain biosynthesis pathway. Simplified representation of the betalain biosynthetic pathway leading to the production of betacyanins and betaxanthins. Enzymes shown are the cytochrome P450 enzymes CYP76AD1 and CYP76AD6, DOPA 4, 5-dioxygenase (DODA), cyclo-DOPA 5-O-glucosyltransferase (cDOPA 5GT), betanidin 5-O-glucosyltransferase (Betanidin 5GT), and betanidin 6-O-glucosyltransferase (Betanidin 6GT). The condensation reactions of betalamic acid with amino acids/amines to form betacyanin and betaxanthin pigments occur spontaneously. [file Data_Sheet_1.zip › Supplementary Figure 7.TIF]

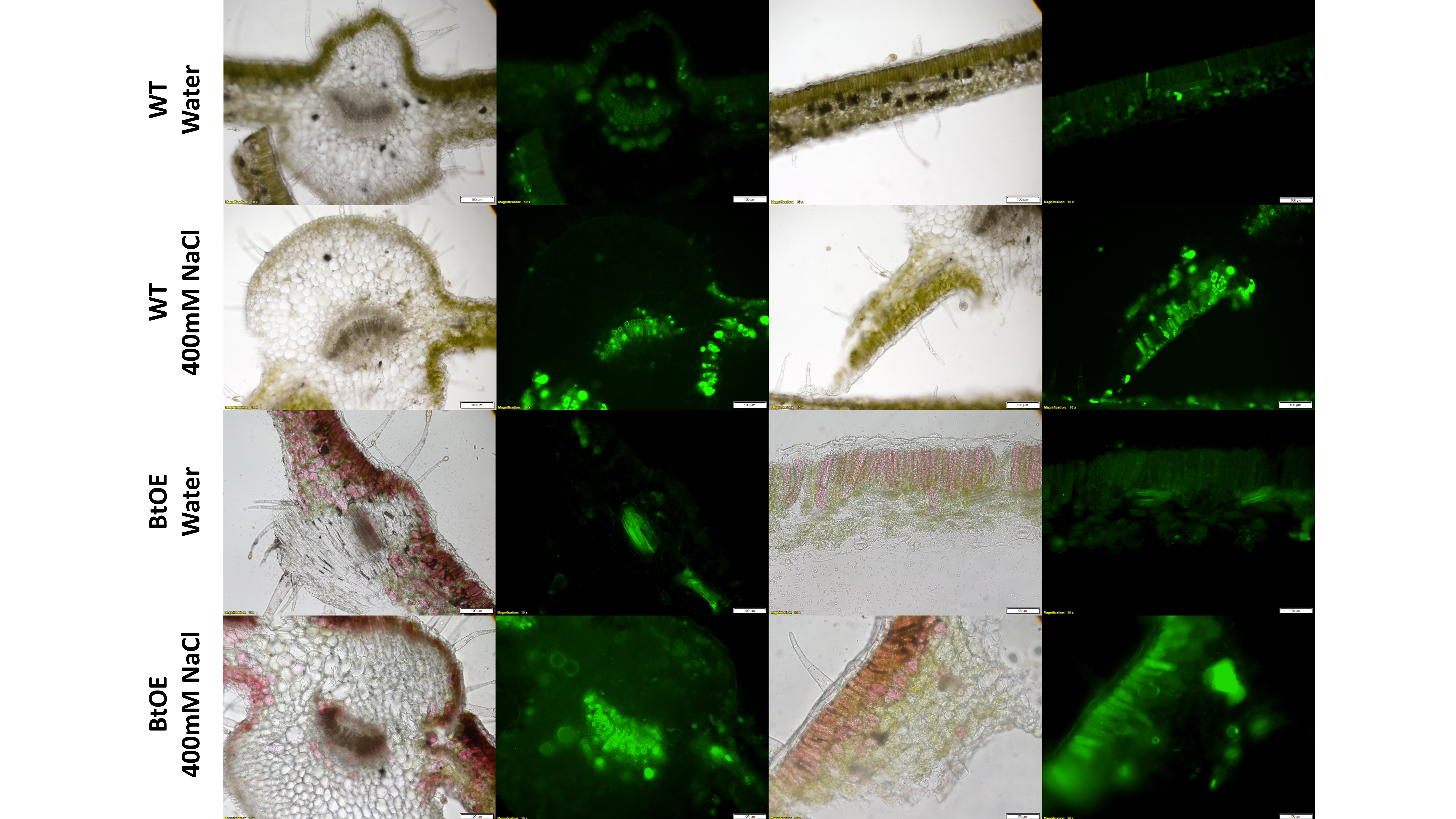

Supplement: Supplementary Figure 1 — The betalain biosynthesis pathway. Simplified representation of the betalain biosynthetic pathway leading to the production of betacyanins and betaxanthins. Enzymes shown are the cytochrome P450 enzymes CYP76AD1 and CYP76AD6, DOPA 4, 5-dioxygenase (DODA), cyclo-DOPA 5-O-glucosyltransferase (cDOPA 5GT), betanidin 5-O-glucosyltransferase (Betanidin 5GT), and betanidin 6-O-glucosyltransferase (Betanidin 6GT). The condensation reactions of betalamic acid with amino acids/amines to form betacyanin and betaxanthin pigments occur spontaneously. [file Data_Sheet_1.zip › Supplementary Figure 8.TIF]
